# Mapping knowledge landscapes and research frontiers of gastrointestinal microbiota and bone metabolism: a text-mining study

**DOI:** 10.3389/fcimb.2024.1407180

**Published:** 2024-07-10

**Authors:** Haiyang Wu, Zaijie Sun, Qiang Guo, Cheng Li

**Affiliations:** ^1^ Department of Orthopaedics, The First Affiliated Hospital of Zhengzhou University, Zhengzhou, China; ^2^ Department of Clinical College of Neurology, Neurosurgery and Neurorehabilitation, Tianjin Medical University, Tianjin, China; ^3^ Department of Orthopaedic Surgery, Xiangyang Central Hospital, Affiliated Hospital of Hubei University of Arts and Science, Xiangyang, China; ^4^ Department of Spine and Joint Surgery, Tianjin Baodi Hospital, Baodi Clinical College of Tianjin Medical University, Tianjin, China; ^5^ Department of Spine Surgery, Wangjing Hospital, China Academy of Chinese Medical Sciences, Beijing, China; ^6^ Center for Musculoskeletal Surgery (CMSC), Charité-Universitätsmedizin Berlin, Freie Universität Berlin, Humboldt University of Berlin, Berlin Institute of Health, Berlin, Germany

**Keywords:** bibliometrics, hotspots, gastrointestinal microbiota, bone metabolism, CiteSpace, VOSviewer

## Abstract

**Introduction:**

Extensive research efforts have been dedicated to elucidating the intricate pathways by which gastrointestinal microbiota and their metabolites exert influence on the processes of bone formation. Nonetheless, a notable gap exists in the literature concerning a bibliometric analysis of research trends at the nexus of gastrointestinal microbiota and bone metabolism.

**Methods:**

To address this scholarly void, the present study employs a suite of bibliometric tools including online platforms, CiteSpace and VOSviewer to scrutinize the pertinent literature in the realm of gastrointestinal microbiota and bone metabolism.

**Results and discussion:**

Examination of the temporal distribution of publications spanning from 2000 to 2023 reveals a discernible upward trajectory in research output, characterized by an average annual growth rate of 19.2%. Notably, China and the United States emerge as primary contributors. Predominant among contributing institutions are Emory University, Harvard University, and the University of California. Pacifici R from Emory University contributed the most research with 15 publications. In the realm of academic journals, *Nutrients* emerges as the foremost publisher, followed closely by *Frontiers in Microbiology* and *PLOS One*. And *PLOS One* attains the highest average citations of 32.48. Analysis of highly cited papers underscores a burgeoning interest in the therapeutic potential of probiotics or probiotic blends in modulating bone metabolism by augmenting host immune responses. Notably, significant research attention has coalesced around the therapeutic interventions of probiotics, particularly *Lactobacillus reuteri*, in osteoporosis, as well as the role of gastrointestinal microbiota in the etiology and progression of osteoarthritis. Keyword analysis reveals prevalent terms including gut microbiota, osteoporosis, bone density, probiotics, inflammation, SCFAs, metabolism, osteoarthritis, calcium absorption, obesity, double-blind, prebiotics, mechanisms, postmenopausal women, supplementation, risk factors, oxidative stress, and immune system. Future research endeavors warrant a nuanced exploration of topics such as inflammation, obesity, SCFAs, postmenopausal osteoporosis, skeletal muscle, oxidative stress, double-blind trials, and pathogenic mechanisms. In summary, this study presents a comprehensive bibliometric analysis of global research on the interplay between gastrointestinal microbiota and bone metabolism, offering valuable insights for scholars, particularly nascent researchers, embarking on analogous investigations within this domain.

## Introduction

The human gastrointestinal tract harbors nearly 1000 trillion different species of microorganisms, forming a complex ecosystem known as the gastrointestinal microbiota (Schmidt et al., 2018). This microbial consortium constitutes the largest ecosystem within the human body and plays crucial roles in various aspects of human physiology, including gastrointestinal development, metabolic processes, nutrition, inflammatory responses, and immune system maturation (Blanton et al., 2016; Gentile and Weir, 2018; Ruff et al., 2020; Fan and Pedersen, 2021). While the impacts of the gastrointestinal microbiota in these areas have been extensively verified, scientific exploration into its additional effects on various systems and detailed mechanisms is actively ongoing.

Bone metabolism encompasses the processes of bone formation and resorption, with osteoblasts and osteoclasts as central players. Osteoclasts are responsible for bone resorption, while osteoblasts are involved in bone formation. Dynamic equilibrium between these two cell types maintains skeletal homeostasis. In recent years, increasing evidence has indicated widespread involvement of the gut microbiota in signaling pathways related to bone metabolism, closely associated with the occurrence and progression of various bone metabolism disorders (Sánchez Romero et al., 2021; Seely et al., 2021; Basak et al., 2024; Zhang et al., 2024). Disruption of the gut microbiota has been shown to negatively impact bone health by impairing intestinal calcium absorption and modulating the balance of the Osteoprotegerin (OPG)/Receptor Activator for Nuclear Factor-κ B Ligand (RANKL) pathway through regulation of multiple hormone levels, consequently reducing bone strength and quality (Han et al., 2023; Cai et al., 2024). Meanwhile, several probiotic strains, such as *Lactobacillus* and *Bifidobacterium*, have been demonstrated to exert significant anti-osteoporotic effects (de Sire et al., 2022; Li et al., 2024; Zhang et al., 2024). Although research on the therapeutic potential of gut microbiota in osteoporosis and osteoarthritis is still in its nascent stage, most scholars envision gut microbiota intervention as a promising strategy for the diagnosis and treatment of bone metabolic diseases in the future.

In light of these findings, the study of the relationship between gut microbiota and bone metabolism has garnered widespread attention (Sánchez Romero et al., 2021; Seely et al., 2021; de Sire et al., 2022; Han et al., 2023; Basak et al., 2024; Cai et al., 2024; Li et al., 2024; Zhang et al., 2024; Zhang et al., 2024). Numerous studies have endeavored to elucidate the intricate mechanisms through which gut microbiota and their metabolites influence bone formation and remodeling, resulting in a plethora of published works. However, faced with the vast amount of literature, researchers often expend considerable time and effort to keep abreast of the latest developments and research dynamics. While some reviews and Meta-analysis offer summaries of key topics from specific perspectives, they may fall short in providing a comprehensive analysis of the overall research trends in the field (Wu et al., 2021a). Moreover, reviews may not furnish scholars, especially newcomers, with the latest information regarding nations, institutions, research clusters, and collaborative efforts. Consequently, owing to the aforementioned limitations of reviews, bibliometric analysis has emerged as a complementary approach, embraced by the biomedical community (Montazeri et al., 2023).

Bibliometric analysis involves the qualitative and quantitative study of all knowledge carriers, such as literature and patents, using statistical, information science, and mathematical methods. This methodology serves as a vital tool for identifying active research teams and potential collaborators, delineating research hotspots, depicting overall research trends, and pinpointing important frontiers for future exploration in a given research field (Wang et al., 2023; Babar et al., 2024). In recent years, owing to the explosive growth of biomedical literature and the continuous development of various free bibliometric tools such as CiteSpace and VOSviewer software, bibliometric studies have garnered increasing attention in the biomedical domain (Aguilar Ramírez et al., 2022; Montazeri et al., 2023; Cheng et al., 2024). Taking the gut microbiota as an example, previous bibliometric studies have analyzed the relationship between gut microbiota and tumors (Zyoud et al., 2022; Wu et al., 2023), inflammatory diseases (Liu et al., 2023; Zhang et al., 2023), immune system disorders (Ni et al., 2022; Zhang et al., 2023), as well as traumatic diseases (Du et al., 2023; Huang et al., 2023). In the field of bone metabolism, several bibliometric analyses have investigated advancements and hotspots in research areas such as osteoporosis (Wu et al., 2021a; Temel et al., 2022), osteoarthritis (Yang et al., 2022; Xiong et al., 2024), and rheumatoid arthritis (Zhong et al., 2021; Radu et al., 2022), mapping the overall knowledge structure and citation networks of these fields. However, to the best of our knowledge, there is no one literature employing bibliometric methods to analyze research hotspots at the intersection of gastrointestinal microbiota and bone metabolism. To address this research gap, the present study employs various bibliometric tools to analyze relevant literature in this field. The primary objectives of this study are to: (1) analyze the overall trends in publications in the field from 2000 to 2023; (2) identify major contributors, including countries, institutions, and funding agencies; and (3) analyze the development and evolution trends of this domain.

## Methods

### Data sources and retrieval strategies

**Figure 1:**
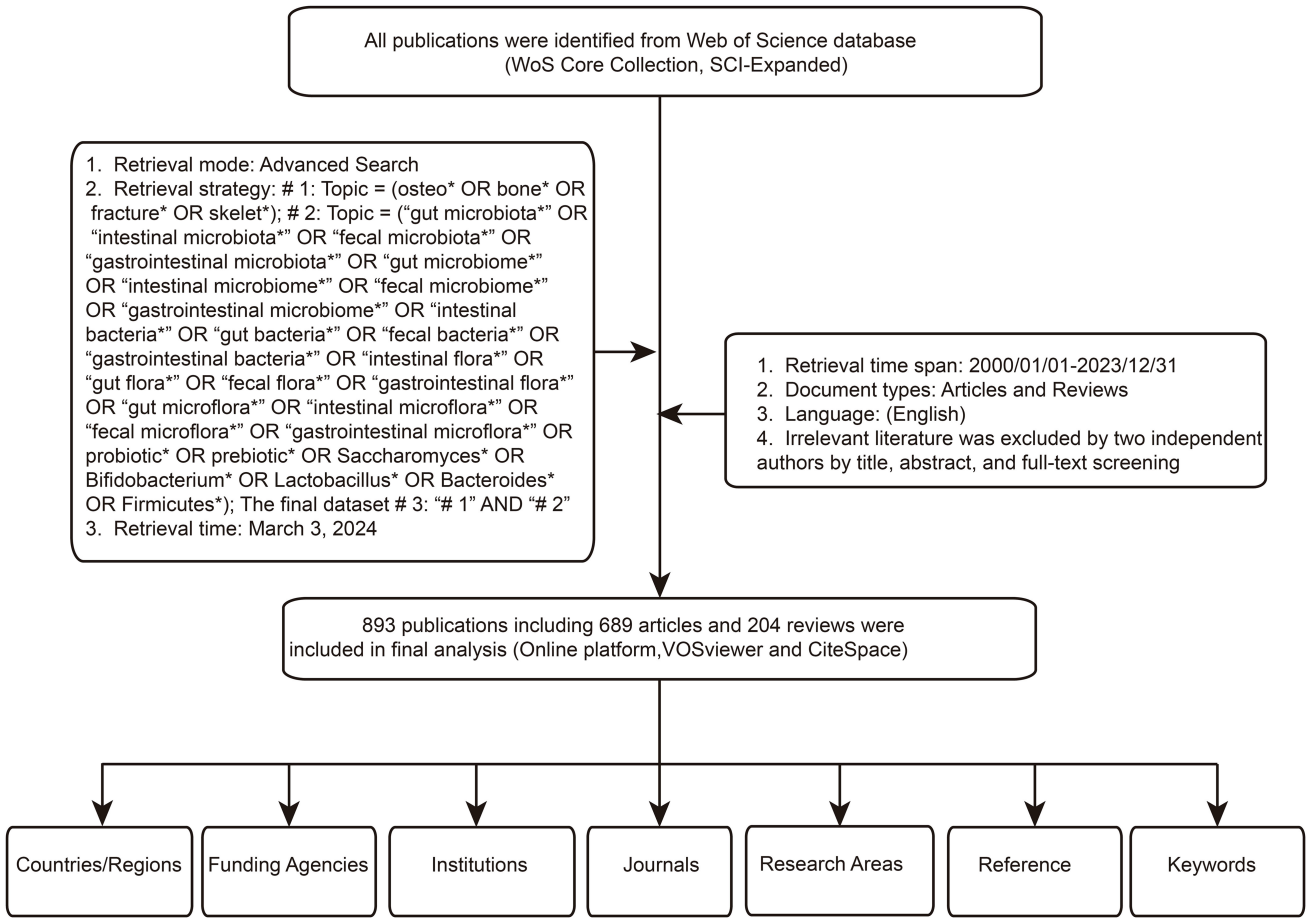
Literature screening process.

In this study, literature retrieval was conducted using the Science Citation Index Expanded (SCIE), a subset of the Web of Science Core Collection (WoSCC), renowned as one of the foremost databases in the biomedical field (Wu et al., 2021a; Aguilar Ramírez et al., 2022; Ni et al., 2022; Temel et al., 2022; Zyoud et al., 2022; Du et al., 2023; Huang et al., 2023; Liu et al., 2023; Montazeri et al., 2023; Wang et al., 2023; Wu et al., 2023; Zhang et al., 2023; Zhang et al., 2023; Babar et al., 2024; Cheng et al., 2024). The database, widely employed in bibliometric studies across disciplines, was accessed on March 3, 2024, with a search spanning a single day. The search strategy employed as follows: # 1: Topic = (osteo* OR bone* OR fracture* OR skelet*); # 2: Topic = (“gut microbiota*” OR “intestinal microbiota*” OR “fecal microbiota*” OR “gastrointestinal microbiota*” OR “gut microbiome*” OR “intestinal microbiome*” OR “fecal microbiome*” OR “gastrointestinal microbiome*” OR “intestinal bacteria*” OR “gut bacteria*” OR “fecal bacteria*” OR “gastrointestinal bacteria*” OR “intestinal flora*” OR “gut flora*” OR “fecal flora*” OR “gastrointestinal flora*” OR “gut microflora*” OR “intestinal microflora*” OR “fecal microflora*” OR “gastrointestinal microflora*” OR probiotic* OR prebiotic* OR Saccharomyces* OR Bifidobacterium* OR Lactobacillus* OR Bacteroides* OR Firmicutes*); The final dataset # 3: “# 1” AND “# 2”. To ensure comprehensive retrieval of relevant literature, wildcard (*) characters were utilized in the search strings to represent variable endings or multiple characters. For instance, “osteo*” could retrieve keywords such as “osteoporotic”, “osteoporosis”, “osteoarthritis”, etc. The publication timeframe was restricted from 2000 to 2023, with language limited to English and document types constrained to articles and reviews. Subsequently, two researchers meticulously reviewed titles, abstracts, and full texts for further screening. The main exclusion criteria were as follows: (1) duplicate literatures; (2) WOSCC sometimes mislabels letter-type papers as articles; manual screening excludes document types other than reviews and articles; (3) Exclusion of research topics unrelated to gut microbiota and bone metabolism. The controversial documents were determined by a third person after careful assessment. Ultimately, a total of 893 articles and reviews was included for final data analysis. **Figure 1** illustrates the literature selection process.

### Data extraction

Following the aforementioned search strategy, the selected literature was downloaded with “Full Record and Cited References” and exported in text or tab-separated format. Microsoft Excel 2019 was employed to count bibliometric metrics such as annual publication counts, citation frequencies, countries/regions, institutions, authors, funding agencies, journals, keywords, and references. The “Citation Report” function in WoSCC was utilized to assess additional bibliometric indicators including total citations, average citations per item (ACI), and the H-index. Journal Impact Factors (IF) and quartile classifications (Q1, Q2, Q3, Q4) were sourced from the 2023 Journal Citation Reports (JCR, http://clarivate.com/products/web-of-science). The H-index is defined as the number of articles (*n*) that have received at least *n* citations. Within the same discipline, JCR categorizes all journals into four quartiles based on their IF, where Q1 represents the top 25%, Q2 the subsequent 25–50%, and so forth. This study also addressed certain inherent deficiencies in the WoSCC database, consolidating and categorizing information from various regions into their respective countries. For instance, publications from England, Northern Ireland, Scotland, and Wales were aggregated under the United Kingdom; while those from mainland China and Taiwan were categorized under China.

### Data analysis

Descriptive data analysis, chart plotting, and curve fitting were performed using Microsoft Excel 2019 and R (v4.1.0). Specifically, Microsoft Excel was employed to visualize trends in annual publication and citation counts, utilizing exponential, linear, logarithmic, or polynomial curve fitting methods, and selecting the optimal model based on the coefficient of determination (R²). The formula for calculating the annual growth rate of publications over time is as follows: Annual Growth Rate = [(Number of publications in 2023 ÷ Number of publications in 2000)^(1/23) - 1] × 100 (Wu et al., 2023). Pearson correlation coefficient tests were conducted to evaluate the correlation between citation counts and publication counts, with a significance level set at *P* < 0.05 indicating statistical significance. Bibliometric and visualization analyses were conducted using three tools: an online bibliometric platform (https://bibliometric.com/), CiteSpace (v6.2R6) (Synnestvedt et al., 2005), and VOSviewer (v1.6.20) (van Eck and Waltman, 2010). CiteSpace, developed by Chen et al., is a widely-used Java-based open-source software for bibliometric analysis. In this study, the parameters for CiteSpace were configured as follows: (1) Time slicing: Each year was used as a time slice from 2000 to 2023; (2) Node types: Keywords and references; (3) Pruning options: Pathfinder, pruning the merged network; (4) Top Nperslice: Set to “Top Nperslice = 30” for author node type and “Top Nperslice = 50” for reference node type. VOSviewer, co-developed by van Eck and Waltman, is another bibliometric software offering text mining capabilities to extract crucial parameters from extensive scientific publications. It provides three types of network maps: network visualization maps, overlay visualization maps, and density visualization maps. The parameters for VOSviewer were configured as follows: type of analysis (select one at a time, such as country/region, institution, journal or keywords), item thresholds (based on specific conditions), and VOSviewer thesaurus file (to merge different variants of keywords). Additionally, this study employed the online bibliometric platform to analyze national collaborations and trends in annual publications.

## Results and discussion

### Trend analysis of publications

**Figure 2:**
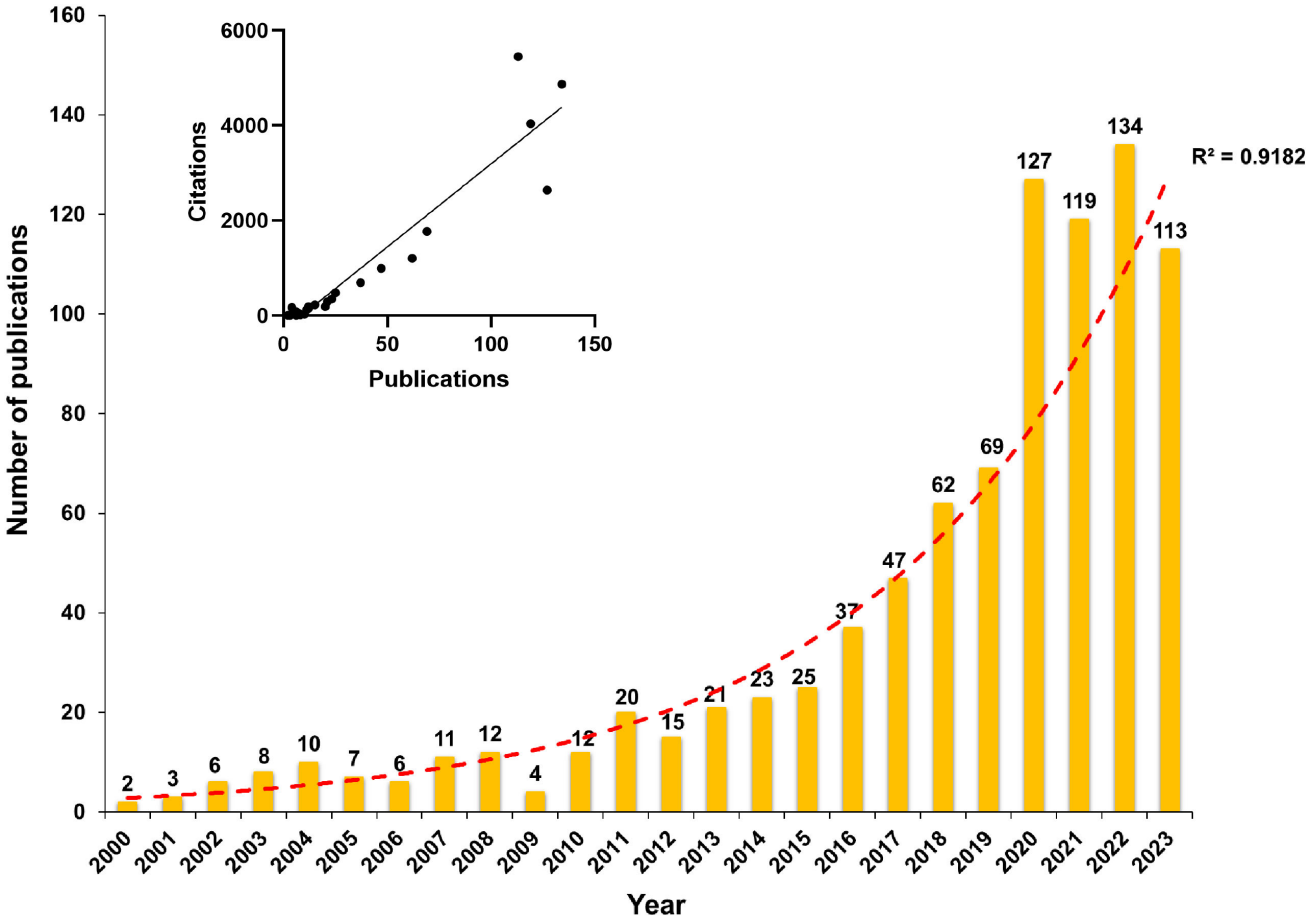
The annual publication trend in the research field of gut microbiota and bone metabolism.

The quantity of publications across different periods directly reflects the developmental trends and transitions within a research domain (Wu et al., 2023). Among the final selection of 893 articles, comprising 689 original articles and 204 reviews, **Figure 2** illustrates the annual distribution of publications in the field of gut microbiota and bone metabolism from 2000 to 2023. It is evident that the overall research output demonstrates a pronounced upward trajectory, with an average annual growth rate of 19.2%, notably surpassing a hundred articles per year in the last four years. In terms of citation frequency, the cumulative total citations for the 893 papers amount to 24658, with an average of 27.61 citations per article. A significant positive correlation exists between the number of publications and citation counts (*r*=0.94, *P*<0.001). These findings indicate a growing interest in the field of gut microbiota and bone metabolism in recent years. This result may be related to the advancements in high-throughput sequencing technologies such as metagenomics and metabolomics (Lau et al., 2023; Hiltzik et al., 2024; Huang et al., 2024). Genomic sequencing of gut microbiota unveils their diversity, abundance, and functional potential, while metabolomics enables scholars to explore the composition and variations of various metabolites within organisms, thereby elucidating the impact of microbiota on host metabolism and the host’s regulation of microbial metabolism (Lau et al., 2023). Previous bibliometric analyses have demonstrated similar trends in research on gut microbiota in areas such as cancer (Zyoud et al., 2022; Wu et al., 2023), inflammatory diseases (Liu et al., 2023; Zhang et al., 2023), immune system disorders (Ni et al., 2022; Zhang et al., 2023), and traumatic injuries (Du et al., 2023; Huang et al., 2023). It is conceivable that with ongoing breakthroughs in omics technologies, our understanding of the relationship and mechanisms between gut microbiota and bone metabolism will be further enhanced, consequently leading to a continued increase in publications within this field in the foreseeable future.

### Analysis of journals and research directions

**Figure 3:**
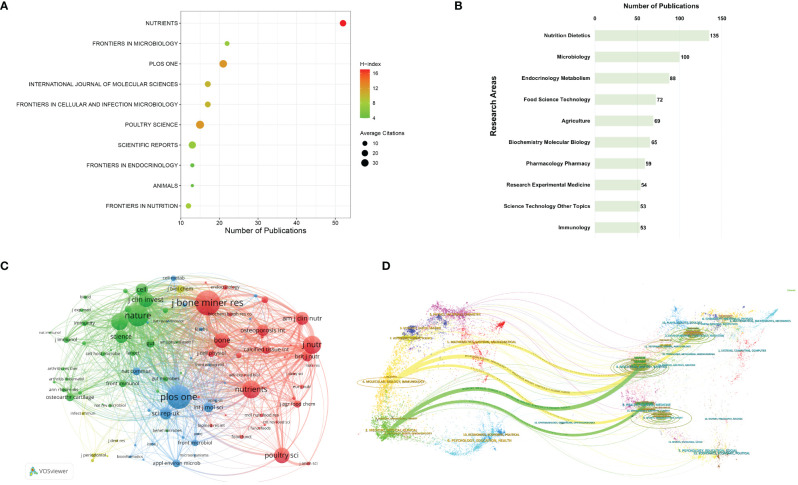
**(A)** The foremost 10 journals in the field of gut microbiota and bone metabolism publications. **(B)** A network analysis of co-cited journals. The size of nodes reflects the cumulative citation counts of the respective journals. **(C)** The top 10 research directions with the highest publication output. **(D)** Dual-map overlay of journals.

For centuries, scientific publications have served as pivotal instruments for scholarly discourse across various domains. Publishing research findings in internationally peer-reviewed journals constitutes a crucial element in establishing effective scientific communication (Wu et al., 2021a). Analyzing major journals within a specific research field could aid researchers in promptly identifying the most suitable outlets and target audiences for their articles. **Figure 3A** summarizes the top10 journals with the highest publication volume. Among these journals, *Nutrients* boasts the highest number of relevant articles, followed by *Frontiers in Microbiology* and *PLOS One*. Most of these journals are classified as Q1 or Q2, with *Nutrients* having the highest IF at 5.9. Regarding the comparison of H-index and ACI, *Nutrients* achieves the highest H-index of 17, while *PLOS One* attains the highest ACI of 32.48. Apart from publication volume, the influence of journals also hinges upon their citation frequency, which is a pivotal determinant of journal IF. In this study, co-citation analysis of journals was conducted using VOSviewer, as depicted in **Figure 3B**. Journals with at least 100 citation times or more were included in the visualization analysis, encompassing a total of 99 journals. Notably, the top 3 co-cited journals are *Journal of Bone and Mineral Research*, *PLOS One*, and *Nature*. The results suggest that these journals have published a considerable volume of high-quality research, garnering substantial attention from scholars in the field.

Furthermore, WoSCC database facilitates the classification of research directions for each article, as illustrated in **Figure 3C**. The top 3 research directions with the highest publication volume are Nutrition Dietetics, Microbiology, and Endocrinology Metabolism. Overall, the findings of this study align with the thematic selection process undertaken.

It is noteworthy that immunology has also emerged as one of the top 10 most scrutinized research directions. In recent years, a growing body of research has unveiled that gut microbiota and their metabolites not only modulate the secretion of endocrine hormones to influence bone remodeling mechanisms but also exert control over bone development by stimulating the immune system (Duque et al., 2011; Wagner and Johnson, 2012; Charles et al., 2015; Hao et al., 2019). For instance, Britton et al. (2014) discovered that *L. reuteri* could suppress the quantity of bone marrow CD4^+^ T lymphocytes, thereby directly inhibiting osteoclastogenesis. Dysbiosis of gut microbiota could promote Th17 cell differentiation, leading to the secretion of inflammatory factors such as IL-1, IL-17a, and tumor necrosis factor α (TNFα), which in turn promotes RANKL generation, inducing monocytes to differentiate into osteoclasts and accelerating bone loss (Luo et al., 2011). Kim et al. (2017) also found that filamentous bacteria isolated from the mouse gut could promote Th17 cell differentiation. Additionally, the dynamic balance between Th-17 and Treg cells serves as a crucial target for gut microbiota. Studies have shown that *Lactobacillus acidophilus* could enhance the secretion of anti-inflammatory factors such as IL-10 and TGF-β by adjusting the Th-17/Treg ratio, thereby reducing osteoclast proliferation and bone resorption (Dar et al., 2018b). Dar et al. (2018a) demonstrated that *Bacillus clausii* could increase Treg cell-promoted bone formation in ovariectomized mouse models. Moreover, Czernik et al. (2021) proved that *Lactobacillus rhamnosus* could promote bone synthesis metabolism by regulating Wnt10b generation mediated by Treg cells. In summary, immune cells serve as a link between gut microbiota and bone metabolism, modulating bone formation or resorption through the regulation of T or B cell functions. With the gradual emergence of the concept of osteoimmunology in recent years, gut microbiota serve as a vital link in exploring the connection between bone and the immune system, offering additional targets for the treatment of bone metabolism disorders (Sharma et al., 2023; Zhivodernikov et al., 2023).

Additionally, as depicted in **Figure 3D**, employing CiteSpace for the overlay analysis of journals enables the visualization of the distribution patterns and citation trajectories of knowledge information across various disciplinary domains represented by citing and cited journals. The thickness of connecting lines signifies the frequency and intensity of information flow between journals. It could be observed that the overlay map of journals in this study exhibits five main information flows. The uppermost yellow flow represents research in the fields of environmental science, toxicology, and nutrition; the middle flow signifies molecular biology and genetics; while the lower flow represents health, nursing, and medical science. These flows converge towards the fields of molecular biology and immunology. Similarly, two additional green flows represent research findings from molecular biology, genetics, health, nursing, and medical science converging towards the fields of medicine, internal medicine, and clinical medicine. The theoretical and technical foundations of research on gut microbiota and bone metabolism originate from these information sources, while the trajectory of information flow depicts the developmental process and evolutionary direction of this field. The convergence points of information flow herald future research frontiers and trends. The results of the overlay graph of journals in **Figure 3D** indicate that future research hotspots in the field of gut microbiota and bone metabolism will focus on molecular biology, immunology, medicine, internal medicine, and clinical medicine.

### Analysis of national and institutional contributions

**Figure 4:**
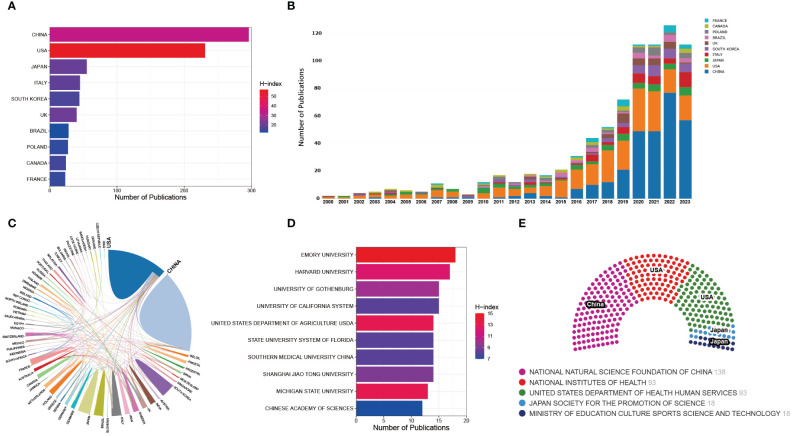
**(A)** Distribution of the top 10 countries in terms of publication output. **(B)** Annual publication trends of the top 10 countries in publication output. **(C)** Analysis of collaboration between countries. **(D)** Distribution of the top 10 institutions in publication output. **(E)** Top 5 funding agencies ranked by funding support.

In this corpus of 893 articles, contributions from 67 countries/regions were identified. Notably, China and the United States emerged as the most prolific contributors, with 296 and 231 publications, respectively, collectively constituting 59% of the total articles. Evidently, both China and the United States stand as primary contributors in this field (**Figure 4A**). Previous studies have underscored the indispensable role of substantial financial support in the advancement of scientific research, highlighting the correlation between the output of scientific research across different countries and their respective Gross Domestic Product (GDP) (Wu et al., 2021b). Consistently, this investigation scrutinized the top 5 funding agencies supporting research in the field of gut microbiota and bone metabolism. The analysis reveals that the National Natural Science Foundation of China (NSFC), the National Institutes of Health (NIH), and the Department of Health and Human Services (HHS) of the United States are the leading sponsors of research endeavors in this domain (**Figure 4E**). This further underscores the correlation between research output from China and the United States and ample funding support. The H-index, defined as the number h of papers that have been cited h or more times, stands as a pivotal metric characterizing both the quality and quantity of research output (Hirsch, 2005). Consequently, this metric serves as a primary indicator for quantifying the productivity and impact of nations or institutions (Dasgupta and Taegtmeyer, 2023; Shah and Jawaid, 2023). According to the H-index, the United States leads with a score of 57, followed closely by China (34) and the United Kingdom (21). Nevertheless, it is worth noting that the H-index is intricately linked with temporal factors, with cumulative citation counts gradually increasing over time for a given study (Wu et al., 2021b). As depicted in **Figure 4B**, the United States dominated the early stages of publications in this field, but in recent years, China has surged ahead, even surpassing the United States in annual publication output by more than half. It can be inferred that the comparatively lower H-index of China, as compared to the United States, may primarily stem from the recent publication of many studies, which have yet to accumulate a sufficient number of citations.

Distinguished research institutions and scholars also play a pivotal role in generating high-quality output. Analysis of the top 10 institutions by publication volume reveals the presence of six American institutions, three Chinese institutions, and one Swiss institution. Notably, the top 3 institutions in terms of publication volume, Emory University, Harvard University, and the University of California, are all situated in the United States (**Figure 4D**), underscoring a potential key factor contributing to the United States’ sustained high-quality output. Furthermore, **Figure 4C** illustrates an analysis of international collaboration, where thicker connecting lines between two countries indicate closer research partnerships. Notably, close research collaboration is evident between China and the United States. In addition, we also we summarized the authors with the highest number of publications in this field. Pacifici R from Emory University contributed the most research with 15 publications (ACI=80.13), followed by Mccabe LR from Michigan State University (ACI=102.36), Ohlsson C from University of Gothenburg (ACI=77.09), Parameswaran N from Michigan State University (ACI=92.91), all of which with 11 publications.

### Analysis of highly cited publications

**Figure 5:**
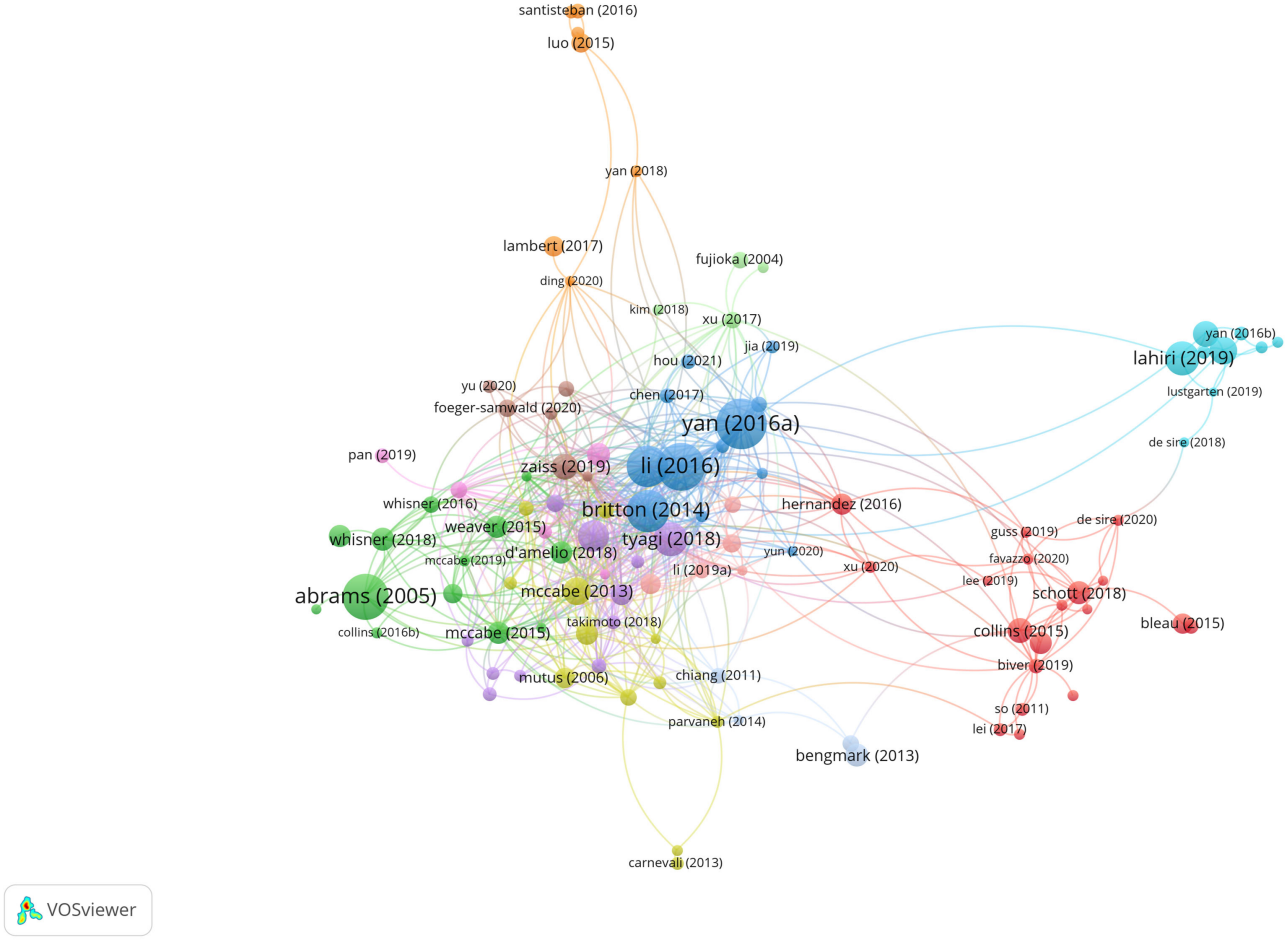
Citation analysis of documents.

Highly cited literature analysis is a commonly utilized method in bibliometric research. While the debate persists regarding whether citation counts entirely represent the impact of a paper, it is generally acknowledged that citation frequency serves as the most objective indicator of research influence within the academic community (Wu et al., 2021a). **Figure 5** illustrates a citation analysis network diagram encompassing literature in gut microbiota and bone metabolism field, with each node representing an article, where node size is proportional to its citation frequency. **Table 1** summarizes the top 10 most cited articles. These studies were published between 2005 and 2019, with 50% of them garnering over 300 citations.

**Table 1:** Top 10 most cited articles.

Ranking	Title	Total Citations	Journal	First Author	Year
1	Gut microbiota induce IGF-1 and promote bone formation and growth	398	*Proceedings of the National Academy of Sciences*	Yan, Jing	2016
2	Sex steroid deficiency-associated bone loss is microbiota dependent and prevented by probiotics	366	*Journal of Clinical Investigation*	Li, Jau-Yi	2016
3	A combination of prebiotic short- and long-chain inulin-type fructans enhances calcium absorption and bone mineralization in young adolescents	348	*American Journal of Clinical Nutrition*	Abrams, SA	2005
4	Probiotic L. reuteri treatment prevents bone loss in a menopausal ovariectomized mouse model	314	*Journal of Cellular Physiology*	Britton, Robert A	2014
5	The gut microbiota regulates bone mass in mice	306	*Journal of Bone and Mineral Research*	Sjogren, Klara	2012
6	The Microbial Metabolite Butyrate Stimulates Bone Formation via T Regulatory Cell-Mediated Regulation of WNT10B Expression	243	*Immunity*	Tyagi, Abdul Malik	2018
7	The gut microbiota influences skeletal muscle mass and function in mice	237	*Science Translational Medicine*	Lahiri, Shawon	2019
8	Probiotics Protect Mice from Ovariectomy-Induced Cortical Bone Loss	217	*Plos One*	Ohlsson, Claes	2014
9	Gut Microbiota Contribute to Age-Related Changes in Skeletal Muscle Size, Composition, and Function: Biological Basis for a Gut-Muscle Axis	187	*Calcified Tissue International*	Grosicki, Gregory J	2018
10	Probiotic use decreases intestinal inflammation and increases bone density in healthy male but not female mice	182	*Journal of Cellular Physiology*	Mccabe, Laura R	2013

Notably, Yan et al. (2016) achieved the highest citation count of 398 for their study published in *PNAS*. Their research revealed that colonization of the gastrointestinal microbiota from conventionally raised SPF mice into germ-free adult mice significantly increased bone formation and bone mass in the latter. Furthermore, they observed a notable elevation in serum IGF-1 levels in germ-free mice following microbial colonization, while antibiotic treatment lowered IGF-1 levels and suppressed bone formation. Supplementation of SCFAs to mice undergoing antibiotic treatment restored IGF-1 levels and bone mass to levels comparable to those of mice not receiving antibiotic treatment. Thus, the authors concluded that the gut microbiota could promote bone formation and growth through the induction of IGF-1.

Ranked second among highly cited literature is the study by Li et al. (2016) published in *J Clin Invest*. This research found that in conventional mouse models, steroid deficiency increased intestinal mucosal barrier permeability, elevated Th17 cells, and upregulated expression of bone resorption factors such as TNFα, IL-17, and RANKL in bone marrow and small intestines, resulting in trabecular bone loss. Conversely, in germ-free mice, steroid deficiency failed to increase bone resorption factor production. Treatment of steroid-deficient mice with probiotic *Lactobacillus rhamnosus* or probiotic supplement VSL#3 significantly reduced intestinal permeability, suppressed bone marrow and intestinal inflammatory factor generation, and completely prevented bone loss. These experimental findings suggest that the gut microbiota serves as a central mediator in the trabecular bone loss induced by steroid deficiency and that methods to reduce intestinal permeability by probiotics may serve as effective therapeutic strategies for postmenopausal osteoporosis.

The third-ranked article is a clinical study that discovered a significant increase in adolescent intestinal calcium absorption and enhanced bone mineralization with daily intake of prebiotic short-chain and long-chain fructo-oligosaccharides (Abrams et al., 2005). Ranking fourth in citation count is a study investigating whether *Lactobacillus reuteri* could mitigate bone loss in an ovariectomized (OVX) mouse model. Results indicated that *Lactobacillus reuteri* could reduce OVX-induced increases in bone marrow CD4+ T lymphocytes and suppress bone resorption. Thus, *Lactobacillus reuteri* treatment may represent an effective approach to treat postmenopausal bone loss (Britton et al., 2014). The fifth-ranked study found increased bone mass and decreased osteoclast numbers in germ-free mice compared to conventionally raised mice, and colonization of germ-free mice with normal gut microbiota restored bone mass, primarily attributed to changes in expression of inflammatory cytokines in mouse bone marrow (Sjögren et al., 2012). While seemingly contradictory to the findings of Yan et al. (2016), both studies concur that the gut microbiota is a critical regulator of mouse bone mass.

Ranked sixth, a study identified that microbial metabolite butyrate stimulates bone formation through Treg cell-mediated Wnt10b expression (Tyagi et al., 2018). The seventh highly cited literature by Lahiri et al. (2019), published in *Sci Transl Med*, observed decreased muscle mass and signs of muscle atrophy in germ-free mice compared to conventionally raised mice. Reduced IGF-1 expression in muscle tissue and significant downregulation of genes associated with skeletal muscle growth and mitochondrial function were noted. Treatment of germ-free mice with SCFAs partially reversed skeletal muscle damage by preventing muscle atrophy and increasing muscle strength. The study underscores the crucial role of the gut microbiota in regulating mouse skeletal muscle mass and function, proposing the concept of the gut microbiota-skeletal muscle axis. The eighth-ranked study found that probiotic treatment could alter skeletal immune status. Specifically, treatment with probiotics such as *Lactobacillus paracasei* reduced expression of inflammatory cytokines TNFα and IL-1β in cortical bone of OVX mice, increased OPG expression, and promoted Treg cell differentiation, thus preventing cortical bone loss (Ohlsson et al., 2014). The ninth-ranked study is a review summarizing the mutual influence between the gut microbiota and skeletal muscle health (Grosicki et al., 2018). Lastly, a study found that *Lactobacillus reuteri* could increase bone formation in male mice by reducing intestinal TNFα levels (McCabe et al., 2013).

In conclusion, current research confirms the close relationship between the gut microbiota and bone/skeletal muscle metabolism, with probiotics and prebiotics regulating bone metabolism by improving host immune status. Moreover, microbiota-based therapies focusing on gut microbiota modulation have emerged as important avenues for treating bone metabolic diseases. In this therapeutic strategy, fecal microbiota transplantation and supplementation with probiotics or prebiotics are garnering significant attention and are extensively researched and discussed.

### Analysis of references

**Figure 6:**
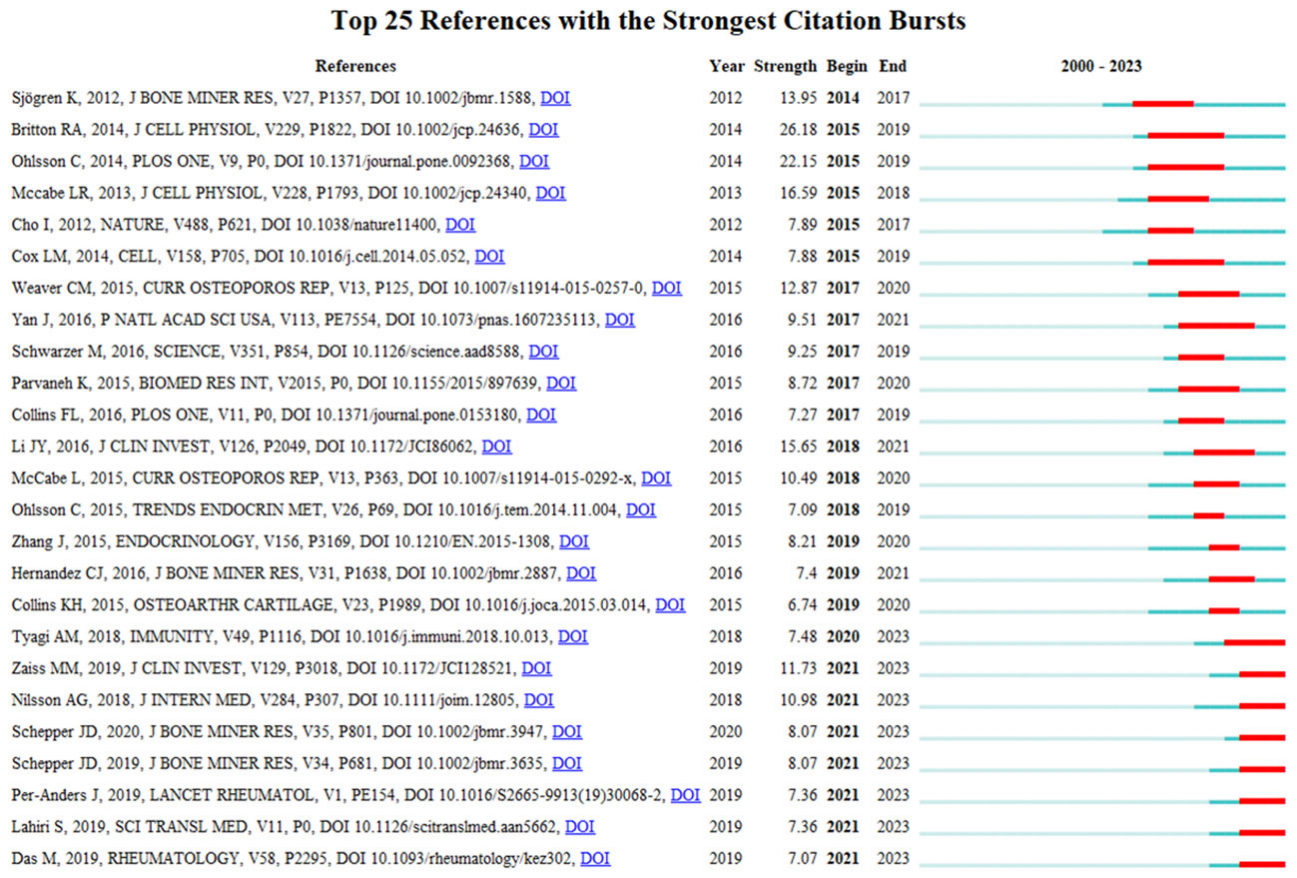
Analysis of reference bursts.

Highly cited literature analysis could only investigate the total citation frequency of papers, but fails to capture the temporal dynamics of attention. The “burst detection” algorithm developed by Kleinberg et al (Kleinberg, 2003). is a commonly used bibliometric method capable of capturing sharp increases in citation attention during specific periods. In this study, we employ the burst detection algorithm to extract citations in the field of gut microbiota and bone metabolism research from 2000 to 2023. **Figure 6** illustrates the top 25 bursting citations. In this figure, the blue lines represent time intervals, while the red lines indicate citation burst periods. Among these citations, the most prominent burst value is associated with a study by Brittond et al. (Britton et al., 2014) published in 2014, which garnered widespread attention from 2015 until 2019, marking a continuous burst period of five years after its publication. As previously mentioned, this study primarily reveals that *Lactobacillus reuteri* could mitigate bone marrow CD4^+^ T lymphocyte expansion induced by OVX, suppress osteoclastogenesis, and reduce bone loss. It is noteworthy that although the burst periods of most citations have concluded, several citations continue to experience ongoing bursts, indicating sustained interest in these research topics in recent years. For instance, Tyagi et al. (2018) confirmed that the microbial metabolite butyrate stimulates bone formation through Treg cell-mediated Wnt10b expression. Additionally, Zaiss et al. (2019) provided a comprehensive review elucidating the crucial regulatory role of SCFAs as metabolites produced by the gut microbiota on the musculoskeletal system and its mechanisms. Nilsson et al. (2018) conducted a randomized, placebo-controlled, double-blind clinical trial to investigate whether *Lactobacillus reuteri* reduces bone loss in elderly women. The study results revealed that daily oral administration of 10^10^ colony-forming units of *Lactobacillus reuteri* for 12 months significantly decreased bone loss in women aged 75 to 80 compared to the placebo, particularly with nearly half the decrease in distal tibial total bone mineral density observed in the placebo group. Schepper et al. (2020) demonstrated that gut microbiota and intestinal barrier function are involved in glucocorticoid-induced osteoporosis, primarily through mechanisms such as Wnt10b inhibition and osteoblast apoptosis, identifying the gut as a novel therapeutic target for preventing glucocorticoid-induced osteoporosis. Another study by Schepper et al. (2019) confirmed that antibiotic treatment results in dysbiosis of the intestinal microbiota and increased intestinal permeability, significantly reducing trabecular bone volume in the femur. *Lactobacillus reuteri*, rather than *Lactobacillus rhamnosus*, prevents antibiotic-induced dysbiosis of the gut microbiota and loss of femoral trabecular bone. This study further emphasizes the role of intestinal microbial dysbiosis-induced changes in intestinal permeability in regulating skeletal health and identifies *Lactobacillus reuteri* as a novel therapy for preventing antibiotic-induced microbial dysbiosis. A randomized, double-blind, placebo-controlled, multicenter trial published by Jansson et al. (2019) in the *Lancet Rheumatology* in 2019 continues to receive attention. The study compared the effects of three probiotics (*Lactobacillus paracasei* DSM 13434, *Lactobacillus plantarum* DSM 15312, and *Lactobacillus plantarum* DSM 15313) combination therapy on lumbar spine bone mineral density in postmenopausal women. The results showed that the combined treatment with the three lactobacilli significantly reduced lumbar spine bone loss in postmenopausal women after 12 months. Das et al. (2019) analyzed fecal microbial profiles of 181 individuals with osteopenia, 60 with osteoporosis, and 60 with normal bone mineral density. The results revealed an association between decreased bone mineral density in individuals with osteopenia and osteoporosis and alterations in the gut microbiota. These changes may serve as biomarkers or therapeutic targets for reducing bone mineral density in high-risk individuals. Summarizing the recent burst of citations, it is evident that probiotic therapy, especially *Lactobacillus reuteri*, for intervention in osteoporosis-related high-quality clinical studies, has garnered significant attention. However, the differential effects of various types of prebiotics or probiotics and whether there are gender, age, and etiological differences in the treatment of osteoporosis remain unclear. Future large-scale, multicenter clinical studies are needed to validate the actual effectiveness of adequate intake of prebiotics or probiotics on human bone health.

**Figure 7:**
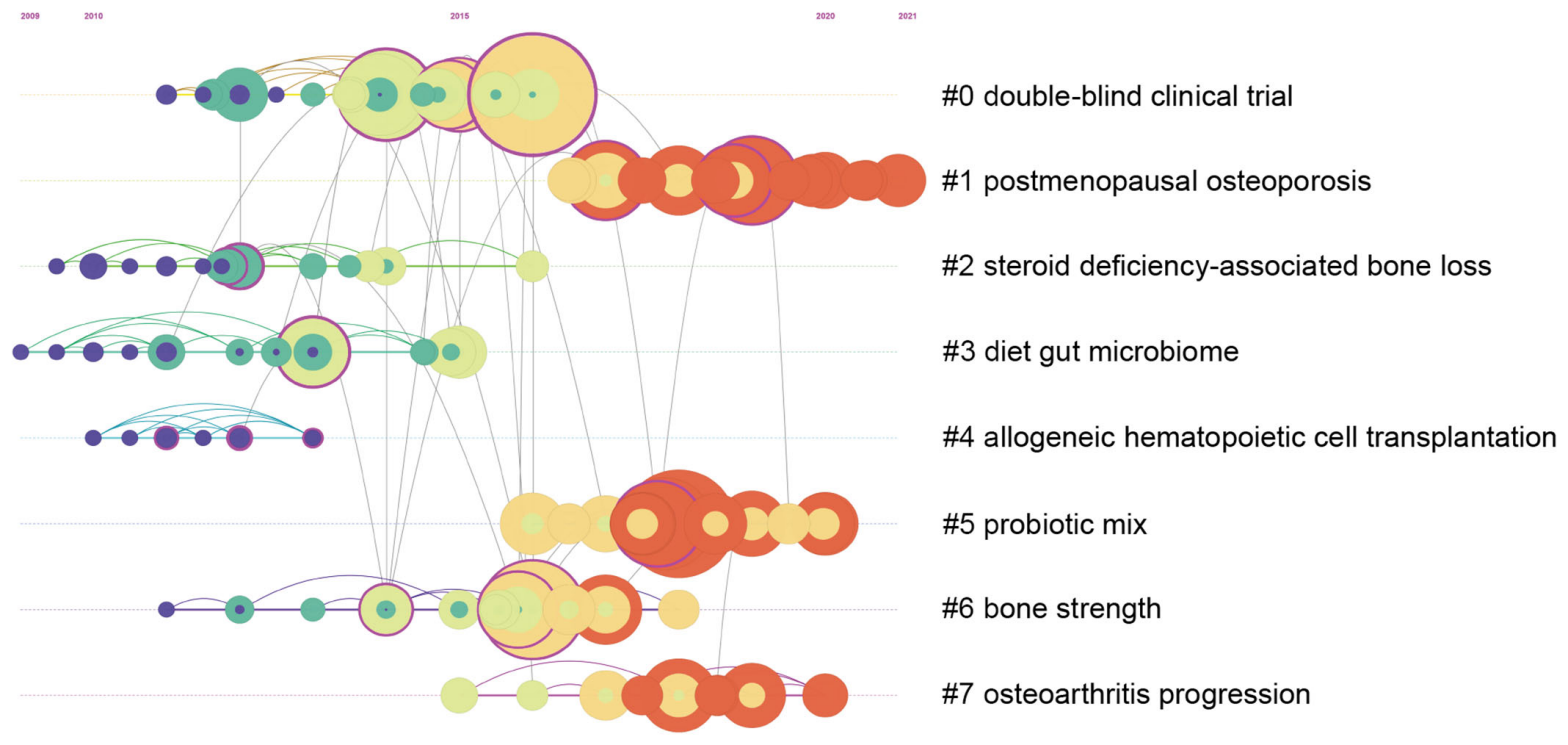
Timeline network map of reference co-citation analysis.

In addition to burst citation analysis, this study also employs CiteSpace software to conduct cluster analysis of citations and arrange the obtained cluster label information in chronological order. The timeline of citation cluster analysis in the field of gut microbiota and bone metabolism is depicted in **Figure 7**. In this figure, cluster labels are named using the log-likelihood ratio (LLR) algorithm, with the homogeneity and modularity parameters being important indicators for assessing the rationality of cluster labels. Both parameters range from 0 to 1, where a Modularity value greater than 0.3 indicates significant modularity, and a Silhouette value greater than 0.7 indicates highly credible clustering effects. In the clusters obtained in this study, Silhouette = 0.9 and Modularity = 0.81, indicating a high degree of homogeneity and modularity in the clusters obtained in this study, suitable for further analysis. Among all cluster labels, those starting with #0 generally contain the highest number of citations, with lower numbers indicating a higher quantity of citations in the cluster. From **Figure 7**, it can be observed that clusters labeled “double-blind clinical trials” and “postmenopausal osteoporosis” occupy the top two positions in terms of the number of citations included, indicating that clinical trials and postmenopausal osteoporosis are the most researched directions in this field. Furthermore, the representation of the timeline intuitively demonstrates the dynamic trends of citation cluster labels over different periods. Based on the average appearance time of different labels, it can be inferred that cluster #1 postmenopausal osteoporosis, #5 probiotic mixtures, and #7 osteoarthritis progression are the hot topics of interest in this field.

In recent years, beyond the established focus on osteoporosis, the role of gut microbiota in the pathogenesis and progression of osteoarthritis has garnered increasing attention (Amin et al., 2023). Substantial research indicates that dysbiosis of the gut microbiota can influence the advancement of osteoarthritis through multiple mechanisms, including the regulation of trace elements (such as iron, zinc, and magnesium), participation in immune responses, and disruption of metabolic processes (Collins et al., 2015; Ulici et al., 2018; Celis and Relman, 2020). For instance, Guss et al. (2019) demonstrated that in a mouse model of osteoarthritis, load-induced cartilage and subchondral bone lesions significantly altered the abundance of Bacteroides and Firmicutes. Similarly, Collins et al. (2015) found notable changes in the abundance of Lactobacillus in the gut microbiota of obese rats induced by a high-fat diet; these changes were significantly associated with levels of inflammatory markers in joint fluid and blood, as well as Mankin scores in the osteoarthritis rat model.

Furthermore, studies have shown that dysbiosis of the gut microbiota can lead to an increase in lipopolysaccharide (LPS)-producing pathobionts, resulting in elevated LPS levels in the bloodstream. This excessive LPS can hyperactivate the immune system, triggering severe inflammatory responses (Arbeeva et al., 2022; Loeser et al., 2022). Previous research has indicated that LPS is directly involved in the osteoarthritis disease process and is significantly correlated with the severity of osteophyte formation, thus suggesting that LPS could serve as a biomarker for osteoarthritis severity (Huang et al., 2016). It can be hypothesized that gut microbiota dysbiosis exacerbates osteoarthritis progression by increasing gut permeability and compromising the intestinal barrier, thereby promoting systemic low-grade inflammation through elevated LPS production.

In addition to inflammatory mechanisms, both clinical and experimental studies suggest that the gut microbiota is a critical factor in metabolic syndrome pathogenesis. Dysbiosis can lead to metabolic disturbances and hormonal imbalances, resulting in conditions such as insulin resistance, hypertension, and central obesity (Croci et al., 2021). Research calculating the combined risk ratios for metabolic syndrome predicting osteoarthritis and vice versa reveals a bidirectional relationship between these conditions (Liu et al., 2020). Consequently, gut microbiota dysbiosis may contribute to osteoarthritis development via metabolic disorder pathways. Given that current research indicates that probiotics, prebiotics, and microbiota transplantation can ameliorate gut microbiota dysbiosis, the gut microbiota presents a promising target for future interventions in osteoarthritis. For instance, Schott et al. (2018) observed that a significant reduction in Bifidobacterium levels in obese mice led to downstream systemic inflammation signals, with macrophages accumulating in the joint synovium, thereby accelerating osteoarthritis progression. Restoring gut microbiota with oligofructose supplementation resulted in decreased systemic inflammation and alleviated arthritis symptoms. Similarly, Sim et al. (2018) demonstrated that intervention with Butyricicoccus in a rat model of knee osteoarthritis significantly reduced serum inflammatory markers such as IL-6 and COX-2 while increasing glycosaminoglycan and IFN-γ levels, effectively reducing fibrotic tissue formation in the knee joint. Other studies have shown that treatment with Bifidobacterium in guinea pig models of osteoarthritis significantly reduced cartilage structure damage and type II collagen degradation (Henrotin et al., 2021). It is important to note that most of these studies are limited to animal models. The efficacy of interventions such as probiotics, prebiotics, and microbiota transplantation in osteoarthritis patients requires extensive clinical trials for confirmation.

### Analysis of keywords

**Figure 8:**
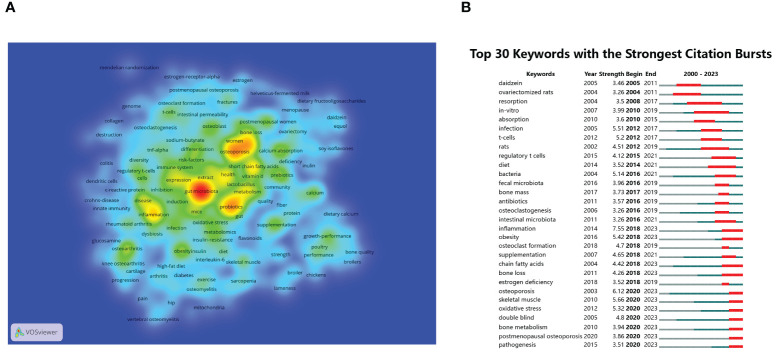
**(A)** Visualization density map of keyword co-occurrence analysis. **(B)** Analysis of keyword bursts.

High-frequency keywords serve as significant indicators of current hotspots in research areas. From a corpus of 893 articles, this study extracted a total of 325 keywords appearing more than 5 times. **Figure 8A** illustrates the co-occurrence map of these high-frequency keywords, where darker colors indicate higher frequency of occurrence. The distribution of keywords reveals several hotspots in research, predominantly centered around gut microbiota, osteoporosis, probiotics, inflammation, and SCFAs. To further quantify the frequency distribution of these keywords, the study summarized the top 20 high-frequency keywords in **Table 2**. Apart from the search term gut microbiota, other popular keywords in this research field include osteoporosis (Biver et al., 2019; Xu et al., 2022; Lyu et al., 2023), bone density (Wang et al., 2023; Zhang et al., 2023b), probiotics (Jia et al., 2021), inflammation (Ding et al., 2022; Zhu et al., 2022), SCFAs (Derrien et al., 2004; Psichas et al., 2015; Whisner et al., 2016; Larraufie et al., 2018; Nath et al., 2018; Shetty et al., 2018; Keirns et al., 2020; Massy and Drueke, 2020; Mohamed et al., 2024; Verstraeten et al., 2024), metabolism (Zhang et al., 2023a), osteoarthritis (Liu et al., 2022; Guan et al., 2023), calcium absorption (Zhou et al., 2023), obesity (Sato et al., 2021), double-blind (Nilsson et al., 2018; Jansson et al., 2019), prebiotics (Mohamed et al., 2024), mechanisms (Zhou et al., 2023), postmenopausal women (Wang et al., 2023), supplements (Li et al., 2023), risk factors (Chen et al., 2021), oxidative stress (Li et al., 2019), and immune system (Kim et al., 2017; Dar et al., 2018b; Dar et al., 2018a). Similarly to citation burst analysis, this study also conducted a burst analysis on keywords that garnered significant attention during a certain period from 2000 to 2023. As depicted in **Figure 8B**, the shifting trends of keywords indicate that earlier studies predominantly focused on *in vitro* mechanistic research, with phrases such as ovariectomy rats, *in vitro*, T cells, and regulatory T cells being highly discussed. Conversely, keywords with bursts extending until 2023 highlight recent hotspots and active research themes, including inflammation, obesity, SCFAs, postmenopausal osteoporosis, skeletal muscle (Li et al., 2024), oxidative stress, double-blind, and pathogenesis, warranting further attention.

**Table 2:** Top 20 high-frequency keywords.

Ranking	Keywords	Occurrence	Ranking	Keywords	Occurrence
1	gut microbiota	454	11	double-blind	56
2	osteoporosis	202	12	prebiotics	53
3	bone mineral density	179	13	bone loss	49
4	probiotics	170	14	mechanism	48
5	inflammation	128	15	postmenopausal women	48
6	short chain fatty acids	108	16	calcium	48
7	metabolism	76	17	supplementation	47
8	osteoarthritis	68	18	risk-factors	46
9	calcium absorption	62	19	oxidative stress	42
10	obesity	61	20	immune system	38

### SCFAs and bone metabolism

Taking SCFAs as an exemplar, SCFAs are a class of metabolic byproducts generated during carbohydrate fermentation by gut microbiota, primarily comprising acetate, propionate, and butyrate. These SCFAs, products of microbial metabolism, such as acetate produced by *Akkermansia muciniphila* in the colonic mucosal layer, and butyrate fermentation by bacteria like *Eubacterium hallii* and *Faecalibacterium prausnitzii* (Derrien et al., 2004; Shetty et al., 2018; Verstraeten et al., 2024), have been shown in previous studies to impact bone metabolism through various pathways. For instance, SCFAs can lower intestinal pH, regulate the expression of calcium transport proteins in intestinal epithelial cells, and facilitate calcium absorption (Whisner et al., 2016). Acetate or butyrate salts produced by gut microbiota can also enhance the expression of calcium-binding protein D9k, significantly promoting intracellular calcium utilization (Nath et al., 2018). Furthermore, SCFAs participate in the secretion regulation of relevant hormones (IGF-1 and glucagon-like peptide-1) to modulate osteoblast and osteoclast differentiation. As previously mentioned, Yan et al. (2016) found that after conventional mice were intervened with broad-spectrum antibiotics or vancomycin, the concentration of SCFAs in the cecum significantly decreased, accompanied by a notable reduction in IGF-1 levels. However, supplementing mice with SCFAs restored circulating IGF-1 levels to normal, indicating a significant role of SCFAs in increasing serum IGF-1. Psichas et al. (2015) discovered that injecting acetate into the colon of wild-type mice significantly increased glucagon-like peptide-1 levels, whereby SCFAs mainly interact with G protein-coupled receptors FFAR2 and FFAR3 on enteroendocrine L cells to promote glucagon-like peptide-1 secretion. It is worth noting that an increasing body of research has found that SCFAs possess immunomodulatory functions, influencing bone metabolism through T and B cell immune mechanisms (Keirns et al., 2020; Massy and Drueke, 2020; Mohamed et al., 2024). On one hand, SCFAs could significantly promote differentiation of intestinal TH17 cells and secretion of various inflammatory factors to induce osteoclastogenesis. On the other hand, SCFAs could induce CD4^+^ T cells to differentiate into Treg cells, activating the Wnt signaling pathway in osteoblasts to promote bone formation. In summary, SCFAs could regulate bone metabolism through various pathways such as modulating intestinal calcium absorption, endocrine pathways, and immune regulatory mechanisms. However, there is still some controversy regarding the mechanisms by which SCFAs promote osteoblast differentiation, with some studies suggesting that SCFAs can promote glucagon-like peptide-1 secretion, while others suggest they can inhibit it (Psichas et al., 2015; Larraufie et al., 2018). Further *in vivo* and *in vitro* experiments are needed to elucidate the regulatory mechanisms of SCFAs on bone metabolism.

### Anti-osteoporosis drugs and gastrointestinal microbiota

In addition, the effects of anti-osteoporosis drugs on the gut microbiota are complex and multifaceted. In recent years, an increasing number of studies have focused on the interactions between bone metabolism and the gut microbiota, as well as the role anti-osteoporosis drugs play in this process. Previous studies have found that certain drugs, such as cinnamic acid (Hong et al., 2022), chondroitin sulfate calcium complex (Shen et al., 2021), and Yigu decoction (Zhang et al., 2023), can increase the number of beneficial bacteria, thereby improving gut health and promoting bone health by reducing the production of inflammatory mediators. Taking chondroitin sulfate calcium complex as an example, it is a commonly used drug for the treatment of bone and joint diseases. Shen et al. (2021) found that intervention with chondroitin sulfate calcium complex could alleviate osteoporosis caused by estrogen deficiency. This effect may be associated with the treatment’s ability to increase the abundance of Acidobacteria, Chloroflexi and Gemmatimonadetes, while decreasing the abundance of Bacteroidetes, Actinobacteria and the B/F ratio at phylum level, along with changes in specific gut microbiota communities at the genus level. However, most studies remain at the animal testing stage, and more clinical cohort studies are needed in the future to confirm these findings. Therefore, more large-scale, high-quality clinical cohort studies are needed in the future to verify the impact of these drugs on human gut microbiota and bone health. Additionally, researchers need to explore the optimal dosage, methods of administration, and potential side effects of these drugs to ensure their safety and efficacy in clinical applications.

## Limitations

The limitations of this study are mainly as follows. First of all, similar to other bibliometric studies, data for this analysis were retrieved only from the WoSCC other than databases like Scopus, PubMed, or Google Scholar. Previous studies showed that WoSCC was the most authoritative and commonly used database with high reliability for bibliometric studies (Jin et al., 2023; Wan et al., 2023). Moreover, recently published high-quality papers may not be identified because they have not received enough citations, which may lead to discrepancies between insights from bibliometric analysis and ongoing real-world advancements.

## Conclusion

Overall, the gut microbiota and its metabolites are closely related to bone metabolism, exerting influences on both osteoblastic and osteoclastic differentiation through various pathways. Further exploration of the impact mechanisms of gut microbiota on bone metabolism could provide more therapeutic targets for a variety of bone metabolic diseases. In this study, we conducted, for the first time, a comprehensive bibliometric analysis of the overall knowledge framework and research status in the field of gut microbiota and bone metabolism. Among the 893 articles finally selected, the trend analysis of annual publication volume clearly indicates that this field is attracting increasing attention. Regarding major contributors, China and the United States undoubtedly dominate, mainly reflected in publication quantity and H-index. The top 3 institutions in terms of publication volume are Emory University, Harvard University, and the University of California. The journal *Nutrients* has the highest number of relevant publications, followed by *Frontiers in Microbiology* and *PLOS One*. Moreover, the *Journal of Bone and Mineral Research*, *PLOS One*, and *Nature* are the most influential journals in this field. As for research directions, Nutrition Dietetics, Microbiology, and Endocrinology Metabolism are the top three directions in terms of publication volume. Analysis of highly cited literature and citation analysis results indicate that the current research focus is on the use of probiotics or probiotic mixtures to regulate bone metabolism by improving host immune status. Probiotic therapy, especially *Lactobacillus reuteri* intervention for osteoporosis treatment, and the role of gut microbiota in the onset and progression of osteoarthritis have received considerable attention. Keyword analysis results reveal that current hot topics mainly include osteoporosis, bone density, probiotics, inflammation, SCFAs, metabolism, osteoarthritis, calcium absorption, obesity, double-blind, prebiotics, mechanisms, postmenopausal women, supplements, risk factors, oxidative stress, and immune system. Future research themes worthy of further attention include inflammation, obesity, SCFAs, postmenopausal osteoporosis, skeletal muscle, oxidative stress, double-blind trials, and etiological mechanisms. In conclusion, this study provides a comprehensive bibliometric analysis of gut microbiota and bone metabolism research from a global perspective, offering valuable reference data for scholars, particularly young researchers, engaged in similar studies in this field.

## Data availability statement

The original contributions presented in the study are included in the article/supplementary material. Further inquiries can be directed to the corresponding authors.

## Author contributions

HW: Conceptualization, Formal analysis, Investigation, Methodology, Software, Writing – original draft. ZS: Conceptualization, Data curation, Investigation, Methodology, Software, Validation, Writing – original draft. QG: Data curation, Methodology, Software, Supervision, Writing – review & editing. CL: Conceptualization, Funding acquisition, Supervision, Writing – review & editing.
